# The Implementation Process of Two Evidence-Based Protocols: A Spatial Neglect Network Initiative

**DOI:** 10.3389/frhs.2022.839517

**Published:** 2022-06-23

**Authors:** Kimberly Hreha, A. M. Barrett, Robert W. Gillen, Chris Gonzalez-Snyder, Jenny Masmela, Peii Chen

**Affiliations:** ^1^Division of Occupational Therapy Doctorate, Department of Orthopaedic Surgery, School of Medicine, Duke University, Durham, NC, United States; ^2^Atlanta VA Health Care System, U.S. Department of Veterans Affairs, Center for Visual and Neurocognitive Rehabilitation, Decatur, GA, United States; ^3^Department of Neurology, Emory University School of Medicine, Atlanta, GA, United States; ^4^Neuropsychology Department, Sunnyview Rehabilitation Hospital, Schenectady, NY, United States; ^5^Division of In-Patient Rehabilitation, Select Medical, Mechanicsburg, PA, United States; ^6^Center for Stroke Rehabilitation Research, Kessler Foundation, West Orange, NJ, United States; ^7^Department of Physical Medicine and Rehabilitation, New Jersey Medical School, Rutgers University, Newark, NJ, United States

**Keywords:** spatial neglect, Prism Adaptation Treatment, knowledge translation, Kessler Foundation Neglect Assessment Process, Consolidated Framework for Implementation Research

## Abstract

**Introduction:**

Spatial neglect, a neurocognitive disorder of lateralized spatial attention, is prevalent among stroke survivors especially in inpatient rehabilitation facilities (IRFs). The ultimate goal of the project was to improve spatial neglect care in inpatient rehabilitation and trained as many OTs as possible using both tools in their regular practices as the means to achieve our overall objective. Therefore, we conducted a project aimed at implementing two evidence-based protocols, one for assessment (KF-NAP®) and the other for treatment (KF-PAT®), and share the implementation process, which included barriers and facilitators identified during and after the process, and implementation outcomes.

**Methods:**

Sixteen IRFs were involved. The Knowledge-To-Action Cycle was used to describe the process of knowledge inquiry (training), translating knowledge (implementation) and evaluating the use of knowledge in clinical practice (outcomes). Barriers and strategies were reported using the Consolidated Framework for Implementation Research and identified through a survey, after the study concluded.

**Results:**

Thirty-two therapists at the participating sites were trained to some level of the KF-NAP and KF-PAT. Throughout the project and also once after it finished, different barriers were identified by researchers and clinicians, who then determined together actions to eliminate or minimize the barriers. For example, multiple sites reported: “not having time to train other staff at their hospital due to high patient volume and other responsibilities.”

**Discussion:**

The project shared our implementation process which demonstrated the importance of using implementation methods and incorporating a researcher-clinician partnership, not only for knowledge generation but also knowledge translation. Frequent communications and exchanging information with stakeholders at different levels, may be determinant to the success of each implementation phase. Further research is needed.

## Introduction

Spatial neglect is a neurocognitive disorder that is characterized by the inability to attend to, perceive, and orient to the space that is contralateral to the injured or damaged cerebral hemisphere (1). It affects 20–40% of stroke survivors (2) and individuals with other acquired brain injuries (3). Since the 1980 s, devastating impacts of spatial neglect on rehabilitation progress, functional recovery, community reintegration, and caregiver burden have been demonstrated in various studies conducted by independent research groups around the world (4–10). Furthermore, spatial neglect prolongs inpatient rehabilitation and increases the risks of falls and injuries (5, 8, 10, 11). Treatments and assessments have been developed, examined, and recommended as guidelines, by organizations such as the American Heart/Stroke Association (12), Canadian Stroke Association (13), and the Australian Stroke Foundation (14). Nonetheless, in practice, it has been arbitrary whether individuals with spatial neglect are provided the recommended evidence-based treatment and assessment services. The problem may be related to the hurdles to achieve knowledge translation, dissemination, and implementation (15–18).

The ultimate goal of the wider research project was to improve spatial neglect care. To achieve this wider goal, we sought to implement two evidence-based standardized protocols, one for assessment and the other for treatment. The assessment protocol was the Kessler Foundation Neglect Assessment Process (KF-NAP®), and the treatment protocol was the Kessler Foundation Prism Adaptation Treatment (KF-PAT®). In this article, we aim to report the implementation process, which included barriers and facilitators identified during and after the process, and share implementation outcomes using quantitative and qualitative information.

**Figure 1:**
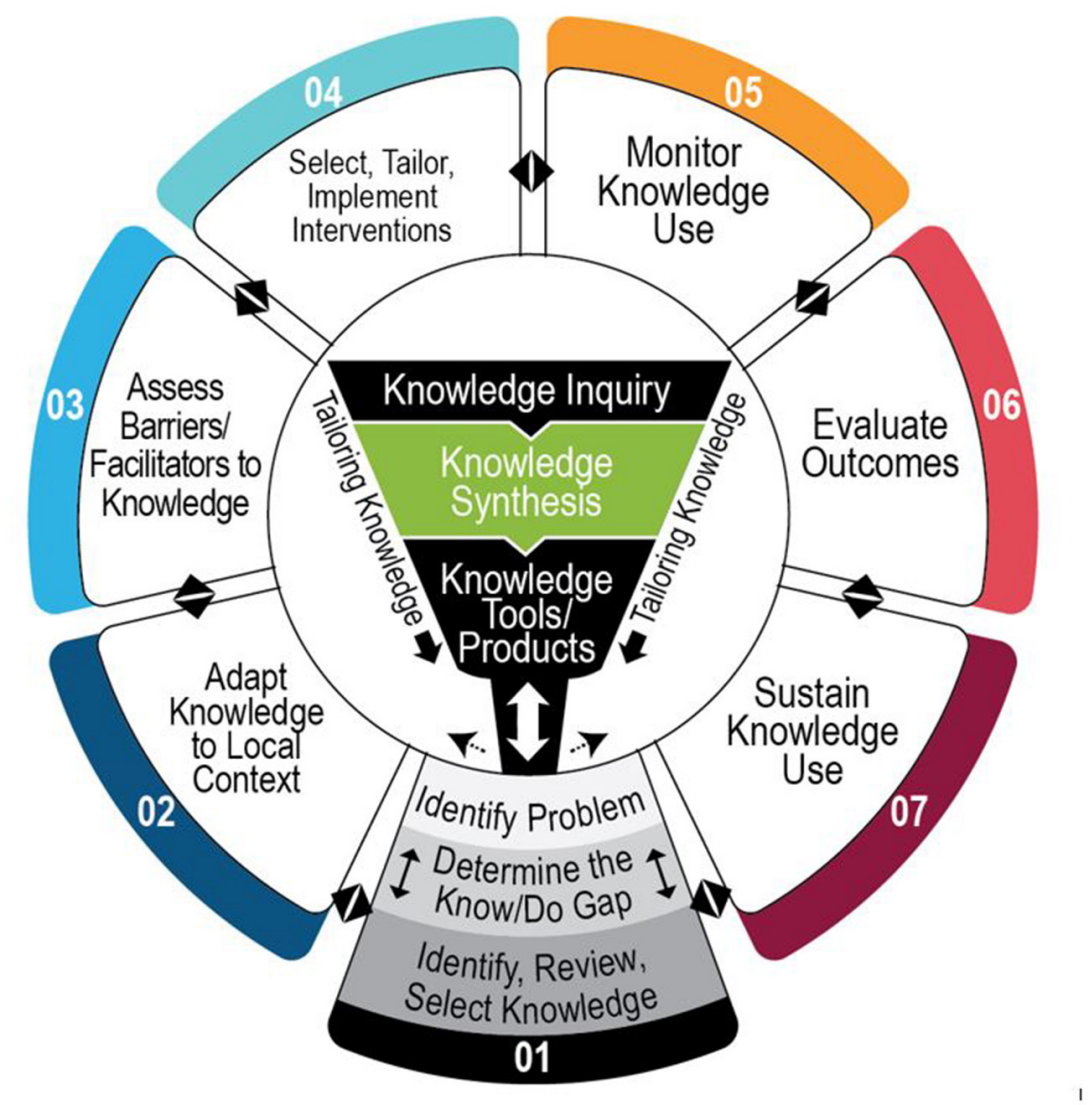
Knowledge-to-action cycle. Adapted from Graham et al. (20) and phases are numbered in this version was our iteration.

In order to report the implementation process systematically, we incorporated methods from the implementation science literature (19). First, we followed the Knowledge-to-Action (KTA) cycle (20) to describe the progression from knowledge dissemination, protocol implementation, to outcome evaluation. The KTA cycle provides a “map” for how to move knowledge into action and encouraged revisiting phases of the action cycle as many times as necessary (Figure 1). The KTA cycle has been widely used in practice because it captures the complexities of real-world application and encourages transformation of knowledge that has been generated in research settings to promote use of evidence-based practices in the clinic (21).

Second, we used the Consolidated Framework for Implementation Research (CFIR), a framework of constructs related to implementation (22), to report barriers identified during the implementation process and the subsequent strategies used to address each barrier (23, 24). The CFIR is organized into five domains (intervention characteristics, outer setting, inner setting, characteristics of the individuals involved and the process of implementation) which provides organization and specificity to evaluate the project's impact (25). There are multiple examples of pragmatic research projects that use the KTA and the CFIR in a rehabilitation setting. Studies suggest that using the CFIR may increase the replicability and generalizability of study findings (26–28), and the KTA cycle can contribute to positive changes in stroke rehabilitation practices (21).

This project implemented the KF-NAP and the KF-PAT. The KF-NAP is a standardized method to administer and score the 10-item Catherine Bergego Scale (CBS) during daily activities (29). The items include gaze orientation, limb awareness, auditory attention, personal belongings, dressing, grooming, navigation, collisions, meals, and cleaning after meals. The scoring uses a scale between 0 and 3 for each item, with the total score ranging between 0 and 30 (the higher the number, the more severe the neglect is). The KF-PAT is a standardized protocol to deliver prism adaption treatment (PAT) (30). PAT is one of the treatment approaches recommended for stroke rehabilitation by the latest guidelines of the American Heart/Stroke Association (12). Both the KF-NAP and KF-PAT, and related materials such as clinician-oriented manuals and equipment, were developed through clinical research and trials over the past decade by our research team (31–35) and thus were the preferred choices in the present implementation project.

The discipline that participated in this project was occupational therapy (OT). Conventionally, in the United States, OT is the discipline in neurorehabilitation providing care related to visuospatial deficits, and is the discipline known to document the observable symptoms of spatial neglect, such as head and eye deviation. Thus, the present project was focused on integrating the two evidence-based protocols into inpatient OT (31–35). In addition, both the KF-NAP and KF-PAT were developed in and for the inpatient rehabilitation care through studies and clinical trials with much involvement of occupational therapists (OTs) (31–35). In the present project, we trained as many OTs as possible using both tools in their regular practices as the means to achieve our overall objective (i.e., to implement both tools) and move closer to our ultimate goal (i.e., to improve spatial neglect care).

## Materials and Methods

### Participants and Setting

Sixteen inpatient rehabilitation facilities (IRFs) across 11 different states in the United States participated in this assessment and treatment implementation project through an agreement with the research center in New Jersey. The agreement included OT training and de-identified clinical information sharing. Twelve sites were on the East Coast (New Jersey, Ohio, Florida, Pennsylvania, Georgia, Maryland, New York), one on the West Coast (California), two in the Southwest (Texas, Arizona) and one in the Midwest (Missouri). The project was approved by the research center's Institutional Review Board (IRB) and the local IRB of each hospital that had a research infrastructure. IRFs without a research infrastructure were attached to the research center's IRB protocol through a federal assurance agreement. Directors of rehabilitation at each site nominated one or two lead OTs (i.e., implementation champions) for project participation. A total of 32 champions were trained to use the KF-NAP and KF-PAT throughout the project. They implemented the protocols in their practice, participated in monthly calls, tracked information regarding implementation, provided feedback to the research center, and were encouraged to train their peers. The implementation information being tracked included de-identified patient clinical records. Patient outcomes were reported separately (36, 37).

The project was initiated in June 2017, and the therapist user feedback completed in March 2021 marking the end of the project. OTs at three sites that had participated in our previous research (31–35) and KF-NAP and KF-PAT development were more familiar with either tools than OTs at other sites. OTs at sites that joined the project earlier might be more experienced with either tool than OTs at sites that joined the project later over the years. While the project was not designed as a research study, lead OTs served as “study participants,” representing their sites, in the user feedback survey at the end of project, and the consent form was waived. Evaluation of the implementation outcomes was based on responses to the survey and information shared about de-identified clinical records.

### Procedures

Following the KTA cycle (Figure 1), the research team led and was actively involved with hospital management leaders and clinicians in the knowledge dissemination (center of the KTA cycle), protocol implementation (Phase 1 to Phase 5), and outcome evaluation (Phase 6). ***Knowledge*** here refers to the two protocols of the KF-NAP and the KF-PAT. Phase 6 required the research team to step back and conduct outcome evaluations. This project did not move into Phase 7.

### Knowledge Dissemination

Knowledge dissemination involved the lead OTs participating in a formal 2-day training. The training took place at either the research center, the IRF where the OTs worked, or a specified IRF that would host a few groups of OTs at the same time. Sixty percentage of the lead OTs received the training at their sites, and the remaining 40% traveled to the location where the training was provided. Completing the training would enable them to reach Level 2 of competency on both protocols (Table 1), and they were instructed and encouraged to fulfill the requirements for the highest level of competency (Level 3) on their own. Level 3 competency would qualify the therapists to be able to teach their colleagues how to administer the KF-NAP or KF-PAT. This entailed using Table 1's criteria to guide the training process. For example, the trainer scheduled time for each therapist to first observe them completing each protocol, and then had the trainees perform the assessment and treatment protocols under supervision.

**Table 1:** Competency levels and criteria.

**Level**	**KF-NAP**	**KF-PAT**
Level 1	Competence to administer the KF-NAP. • Observing Trainer's administration with at least 1 patient. • Creating the environment for KF-NAP. • Assessing the 10 functional activities in no more than one visit.	Competence to administer the KF-PAT. • Having read the KF-PAT Manual. • Observing Trainer's administration with at least 1 patient. • Under Trainer's instruction and supervision, performing at least 1 session with an actor patient.
Level 2	Competence to score the CBS following the KF-NAP. • Observing Trainer's scoring with at least 1 patient. • Scoring at least 2 patients with Trainer's supervision.	Competence to treat patients using the KF-PAT. • Under Trainer's supervision, performing at least 2 sessions with patients.
Level 3	Competence to train other therapists to use the KF-NAP. • Having assessed and scored at least 10 patients independently. • Creating the environment for KF-NAP in a novel environment (e.g., examination room).	Competence to train other therapists to use the KF-PAT. • Having treated at least 5 patients independently.

The 2-day training was taught by the same members of the research team, who developed the protocols and have therefore extensive knowledge on the topic of spatial neglect. The curriculum started with didactics about spatial neglect and then the two protocols, hands-on practice using both protocols under the trainer's supervision and ended with discussions on implementation. The lead OTs were provided training materials including the Manuals, lecture handouts, and pre-recorded video tutorials. Fidelity of both the assessment and intervention were discussed when these materials were being distributed. This discussion included that the Manuals were mandatory to use, the training of other staff members must include the lecture handouts, video tutorials should be reviewed prior to the in-person supervision and hands-on-training, and competency forms should be used. The lead OTs were also instructed about how the ways in which to communicate with the research team.

In addition, the research team offered lectures to other disciplines of therapists, medical staff, case managers, and trainees during new employee trainings, regular staff meetings, and schedule meetings outside of working hours. The lectures focused on the mechanisms, clinical presentations, and consequences of spatial neglect. These knowledge dissemination activities were developed to help coworkers of the OTs understand why the KF-NAP and KF-PAT were being implemented in their facilities. Thus, consistent with “culture,” a construct of the inner setting domain within the CFIR framework, an environment friendly for spatial neglect care was cultivated, potentially beneficial for the implementation project.

### Implementation Process

#### Daily Practice and Frequent Communications

After the training, the lead OTs returned to their daily clinical activities and started to gain experience by completing the protocols independently with their own patient case load. They were instructed to assess all neurological patients for spatial neglect using the KF-NAP within 4 days after admission, regardless of whether patients' symptoms were clearly observable or not. In addition, the lead OTs were also instructed to treat patients using the KF-PAT Portable Kit^1^ when spatial neglect was confirmed through the KF-NAP, and assess these patients using the KF-NAP again after completing 10 sessions of PAT or before IRF discharge.

In addition to implementing both the KF-NAP and the KF-PAT in their clinical practice, the lead OTs were asked to document why the assessment and treatment sessions were not performed or performed in a way deviated from the standardized manuals. The research team provided a spreadsheet template to the lead OTs and required that it be filled out with de-identified clinical records of patients who were assessed using the KF-NAP and who were treated using the KF-PAT. The spreadsheet was submitted to the research team every quarter. This spreadsheet was one way that the barriers to implementation and strategies trialed were communicated. The other way was during the monthly calls.

The research team hosted monthly 1-h conference calls with all participating IRFs, represented by the lead OTs. This is an example of the KTA cycle being used to monitor outcomes. A total of 37 conference calls were conducted from June 2017 to September 2020, and they were recorded to document the meeting and later used to create meeting minutes. These minutes were shared via email to all attendees and the lead OTs who were not able to attend. During the calls, the OTs and the research team had the opportunity to share progress related to the training and implementation. The OTs shared any facilitators or barriers implementing either protocol that they may have listed in their spreadsheet or that were new and therefore being reported for the first time. For example, a facilitator was that OTs exchanged experiences about obtaining leadership support when integrating both protocols into their clinical practice. Barriers often shared were related to managing time and resources. When a barrier was shared, other OTs on the call would offer suggestions that worked or did not work for them, and the research team would help determine a resolution. However, if no resolution was suggested during the call or if the solution suggested was not agreed upon, the research team would follow up with the clinicians and sometimes their supervisors after the call. The OTs were encouraged to contact the research team via email at any time for further questions and comments. The calls also allowed the research team to clarify details in the assessment or treatment protocols that might have been forgotten from the training. In addition, starting at the June 2018 meeting, the research team would ask an OT to share a patient case to the group. The cases presented usually included a short medical history and details of either protocol used with that patient. The attendees could offer their comments and asked questions. The sharing of cases facilitated engagement during the meetings as well as encouraged discussions and comradery.

#### Engaging Other Stakeholders

While the project was focused on integrating the evidence-based protocols into the OT's clinical practice, there were other stakeholders who played significant roles. Because the OTs were involved in the implementation project, there were increased discussions about spatial neglect during care team meetings attended by all disciplines that provided medical and therapy services. In addition, other disciplines were able to easily and informally observe how the KF-NAP and KF-PAT were administered in spaces shared by all disciplines. Also, at several participating sites, the OTs conducted in-services with physicians, nurses, and nutrition staff to share information about this implementation project. This involvement of multiple disciplines could be one action that facilitated the implementation progress, especially in Phase 3 and Phase 4 of the KTA cycle (Figure 1). Also, the IRF administrative leadership were engaged in discussions with the Chief Executive Officers and supported the implementation of both protocols after reviewing implementation goals of the project. Directors of Rehabilitation were informed with the project progress and were encouraged to offer comments and suggestions throughout the project.

#### Addressing Barriers

The implementation procedures evolved and adapted to different hospital contexts as we continuously learned from OTs' experience using either KF-NAP or KF-PAT protocol with patients and interacting with their colleagues, and from administrators' guidance on hospital regulatory and operational standards. From time to time, a situation would occur that was not expected, or a solution was not immediately available, and we classified the situation as a ***barrier***. When barriers were identified, via verbal or written report, then strategies were carefully customized based on the needs of the setting, and then implemented. A strategy and related actions were decided and executed based on the available resources and contextual situations at the time, usually right after the barrier was identified, rather than based on a pre-determined decision-making roadmap. We followed the CFIR framework (22) to organize the identified barriers and summarize strategies and actions taken to eliminate the barriers (Table 2). Barriers were identified in 4 of the 5 CFIR domains. In these domains, there were no identified barriers in some constructs, but a number of barriers in other constructs. Throughout the process, two limiting factors—time and staff—emerged frequently across different constructs and domains. Time was limited against the administration of the KF-NAP and KF-PAT. Shortage of trained staff trained on the two protocols was a common barrier to assessing all patients with neurological conditions and treating patients with spatial neglect. Collaborating with clinicians and their managers, we offered potential solutions. Some of the barriers identified were resolved shortly such as those identified in the Intervention Characteristics domain (Domain I, Table 2), some barriers ultimately required multiple strategies to fully address the problem such as those identified in the Inner Setting domain (Domain III), and others remained challenging such as several barriers identified in the Process domain (Domain V).

**Table 2:** Barrier assessment by the Consolidated Framework for Implementation Research (CFIR) with actions to remove the barrier.

**Domain I:** Intervention characteristics (Key attributes of interventions, i.e., KF-NAP^**®**^ and KF-PAT^**®**^, that influence the success of implementation). No barrier was identified in four constructs including **intervention source** (stakeholder perception about whether the intervention is externally or internally developed), **evidence strengthen and quality** (stakeholder perception of the quality and validity of evidence supporting the belief that the intervention will have desired outcomes), **relative advantage** (stakeholder perception of the advantage of implementing the intervention vs. an alternative solution), and **trialability** (the ability to test the intervention on a small scale in the organization, and to be able to reverse course if warranted).		
**Construct (definition)**	**Barrier**	**Strategy and actions to remove the barrier**
**Adaptability** (The degree to which an intervention can be adapted, tailored, refined, or reinvented to meet local needs)	• KF-NAP assessment: ° Time limitation against adding a new assessment protocol ° Determining best time to complete the assessment during the admission and before discharge • KF-PAT treatment: ° Set-up time ° Patients with varied medical, physical, cognitive, and neuropsychological conditions (also see Domain II: Outer Setting, the construct of Patient Needs and Resources)	• KF-NAP assessment: ° Suggestions made to integrate KF-NAP with conventional ADL assessment in the morning to increase the efficiency of time allocation ° Instruction added to complete the assessment by Day 4 from admission date (giving patients time to be acclimated to the facility) and give enough time for treatment to be complete ° Suggestions made to prioritize KF-NAP assessment after KF-PAT completion or the day before discharge • KF-PAT treatment: ° Solutions provided to reduce the set-up time, such as allowing assistance of therapy aides to set up the equipment and laminating the stimulus sheets. ° Instructions refined and clarified in the manuals for how to provide commands and when to skip a task and move on. ° Suggestion to consult with optometry if the person was prescribed with optical lenses. Reading glasses can be used under the prism lens, if necessary.
**Complexity** (Perceived difficulty of implementation, reflected by duration, scope, radicalness, disruptiveness, centrality, and intricacy, and number of steps required to implement)	• KF-NAP assessment: ° Time limitation against completing all 10 items. • KF-PAT treatment: ° Hesitance of some neuro-optometrists who were unfamiliar with the intervention. ° Time limitation against fitting the treatment into an OT therapy session	• KF-NAP assessment: ° In-person instructor-guided assessment practice to demonstrate how to complete all 10 items in one session. ° Suggested actions to take in order to decrease assessment time based on the facilities' unique needs. • KF-PAT treatment: ° Invitations to neuro-optometrists to discuss the treatment mechanisms and why the KF-PAT is within the scope of OT ° In-person instructor-guided treatment practice to demonstrate how to fit the treatment into regular OT sessions
**Design quality and packaging** (Perceived excellence in how the intervention is bundled, presented, and assembled)	• KF-NAP assessment: ° The booklet of the manual was not easy to carry around when administering the assessment. • KF-PAT treatment: ° Equipment assembly not always intuitive. ° Device not fit on patients with a much smaller or larger body size. ° Frequent wear and tear of the equipment	• KF-NAP assessment: ° A two-page double-side-printed shortened ‘cheat sheet' was developed. • KF-PAT treatment: ° Added pictures to the manual and production of short video clips showing how to set up the equipment. ° The visual field occluder (the wearable shelf) was modified to accommodate a wider range of body sizes. ° Device repairs and replacement were provided.
**Cost** (Costs of the intervention, and costs associated with implementing the intervention, including investment, supply and opportunity costs)	• KF-NAP assessment: ° Limited budget for staff training • KF-PAT treatment: ° Did not have the equipment	• KF-NAP assessment: ° Provision of 100% discount to the online tutorial ° Free email and phone consultation ° Support from the management to provide travel funds for therapists to attend in-person hands-on trainings • KF-PAT treatment: ° The equipment was loaned to the sites by the research team, as part of the research agreement
**Domain II:** Outer setting (The economic, political, and social context within which an organization resides). No barrier was identified in three constructs including **cosmopolitanism** (the degree to which an organization is networked with other external organizations), **peer pressure** (mimetic or competitive pressure to implement an intervention), and **external policies and incentives** (a broad construct that includes external strategies to spread interventions).		
**Construct (definition)**	**Barrier**	**Strategy/action used to remove the barrier**
**Patient needs and resources** (The extent to which patient needs, as well as barriers and facilitators to meet those needs, are accurately known and prioritized by the organization)	• KF-NAP assessment: ° Not all 10 items were scored in certain patients due to physical disabilities or cognitive impairment.	• KF-NAP assessment: ° Solutions and in-person demonstrations regarding how to build a rapport with patients, observe neglect symptoms, and score as many items as possible
	° Some patients refused to comply with the assessment protocol. For example, not feeling comfortable being observed when having a meal	• KF-PAT treatment: ° Solutions and in-person demonstrations regarding how to work with patients with severe neglect symptoms
	• KF-PAT treatment: ° Unable to follow commands due to severe neglect symptoms ° Unable to follow commands due to language barriers in non-English-speaking patients ° Unable to use certain equipment components as intended due to physical disabilities ° Unable to tolerate the prism goggles (e.g., feeling dizzy or seeing doubles due to optical shifts, too much physical pressure on the head by the goggles for patients who wear a helmet)	° Simplified directions and gesturing to work with non-English-speaking patients ° Device modifications and alternative ways of putting on goggles to allow most patients to participate in the treatment
**Domain III:** Inner setting (Features of structural, political, and cultural contexts through which the implementation process will proceed). No barrier was identified in one construct, which was **structural characteristics** (the social architecture, age, maturity, and size of an organization).		
**Construct (definition)**	**Barrier**	**Strategy/action used to remove the barrier**
**Networks and communications** (The nature and quality of webs of social networks, and the nature and quality of formal and informal communications within an organization)	• Miscommunication and confusion with the implementation process • Research documentation including activities completed by the therapists were disorganized • Outdated knowledge on the mechanisms and presentations of spatial neglect. For example, the disorder was often referred by clinicians as “visual neglect” and thus PAT was incorrectly considered a vision therapy	• Standardization of both protocols to establish what must be followed and what can be modified • Solutions provided to improve the organization of information and the quality of the communication • Provision of additional education sessions from the researchers to clarify the principles of KF-NAP and KF-PAT and what spatial neglect is, from neurological and neuropsychological mechanisms to clinical presentations
**Culture** (Norms, values, and basic assumptions of a given organization)	• Profitability unknown	• Production of an information brochure about spatial neglect and KF-PAT treatment, targeted at potential clients (patients and their family members) • New research projects designed to examine to what extent the implementation of KF-NAP and KF-PAT reduces cost while improving quality of care
**Implementation climate** (The absorptive capacity for change, shared receptivity of involved individuals to an intervention, and the extent to which the use of the intervention will be rewarded, supported, expected within their organization). No barrier was identified in three sub-constructs including **compatibility** (the degree of tangible fit between meaning and values attached to the intervention by involved individuals, how those align with individuals' own norms, values, and perceived risks and needs, and how the intervention fits with existing workflows and systems), **goals and feedback** (the degree to which goals are clearly communicated, acted upon, and fed back to staff, and alignment of that feedback with goals), and **learning climate** (a climate in which: leaders express their own fallibility and need for team members' assistance and input; team members feel that they are essential, valued, and knowledgeable partners in the change process; individuals feel psychologically safe to try new methods; and there is sufficient time and space for reflective thinking and evaluation).		
**Sub-construct (definition)**	**Barrier**	**Strategy/action used to remove the barrier**
**Tension for change** (The degree to which stakeholders perceive the current situation as intolerable or needing change)	• Competing demands in the therapy departments, self-initiative of the therapists to use the protocols consistently	• Frequent communications with the IRF management highlighting that these protocols would provide guidance for staff, and the potential impact of the implementation on quality of care
**Relative priority** (Individuals' shared perception of the importance of the implementation within the organization)	• KF-NAP assessment: ° Some therapists prioritized the administration of KF-NAP in patients who already showed neglect symptoms • KF-PAT treatment: ° Some therapists did not provide KF-PAT to patients with “very mild” neglect	• KF-NAP assessment: ° Frequent reminders during staff training and monthly calls that it was of great importance to assess all patients with neurological conditions because certain symptoms were not apparent. It was also important to confirm the absence of spatial neglect.
	° Some therapists prioritized other treatment than KF-PAT in patients whose length of stay was pre-determined to be shorter than 10 days	• KF-PAT treatment: ° Frequently discussed during monthly calls regarding the factors to be considered in initiating and completing the treatment.
**Organizational incentives and rewards** (Extrinsic incentives such as goal-sharing awards, performance reviews, promotions, and raises in salary, and less tangible incentives such as increased stature or respect)	• No competency measure existed as well as no specific incentive to participate in the research project	• Free meal if training lectures were offered during the lunch hour • Development of competency certification processes for both KF-NAP and KF-PAT such that certificates could be added to therapists' profiles, which may help promotion • Participation in the research was an approved task that counted toward clinical promotion
**Readiness for implementation** (Tangible and immediate indicators of organizational commitment to its decision to implement an intervention). No barrier was identified in one sub-construct, which was **access to knowledge & information** (ease of access to digestible information and knowledge about the intervention and how to incorporate it into work tasks).		
**Sub-construct (definition)**	**Barrier**	**Strategy/action used to remove the barrier**
**Leadership engagement** (Commitment, involvement, and accountability of leaders and managers with the implementation)	• A few sites were slow in executing the collaborative agreement with the research team, delaying the initiation of the project • Some sites were under leadership changes during the project • Several sites were at the relatively early stage of hospital development	• Increased frequency of communications with the IRF management • Seeking assistance from the Reginal management team
**Available resources** (The level of resources dedicated for implementation and on-going operations)	• Limited budget for outside staff training • Limited time allocated for lead OTs to train other OTs • No budget to acquire KF-PAT equipment additional to the initial one provided by the research team	• Provision of 100% discount to the online KF-NAP tutorial • Free email and phone consultation • Support from the management to provide travel funds for therapists to attend in-person hands-on trainings • Collaboration between researchers and IRF leaders to improve the staff training capacity for the goal of assessing all neurological patients and treating all patients with spatial neglect with prism adaptation • Working with the IRF management to understand the threshold for capital purchase requests
**Domain IV:** Characteristics of individuals (Characteristics of OTs implementing KF-NAP® and KF-PAT®). No barrier was identified in all five constructs including **knowledge and beliefs about the intervention** (individuals' attitudes toward and value placed on the intervention as well as familiarity with facts, truths, and principles related to the intervention), **self-efficacy** (individual belief in their own capabilities to execute courses of action to achieve implementation goals), **individual stage of change** (characterization of the phase an individual is in, as he or she progresses toward skilled, enthusiastic, and sustained use of the intervention), **individual identification with organization** (a broad construct related to how individuals perceive the organization, and their relationship and degree of commitment with that organization), and **other personal attributes** (a broad construct to include other personal traits such as intellectual ability, motivation, values, competence, capacity and learning style).		
**Domain V:** Process (Essential activities of implementation process). No barrier was identified in one construct, which was **planning** (the degree to which a scheme or method of behavior and tasks for implementing an intervention are developed in advance, and the quality of those schemes or methods).		
**Construct (definition)**	**Barrier**	**Strategy/action used to remove the barrier**
**Executing** (Carrying out or accomplishing the implementation according to plan)	• Initial lead OTs left the position or were on medical leave • Not all OTs were trained to administer the KF-NAP or KF-PAT	• Collaboration between researchers and IRF leaders to improve the staff training capacity ° Training experienced and enthusiastic therapists to become lead OTs and trainers ° Developing remote education modules
**Reflecting and evaluating** (Quantitative and qualitative feedback about the progress and quality of implementation accompanied with regular personal and team debriefing about progress and experience)	• Infrequent feedback provided from therapists who did the frontline work of implementation	• Centralizing the information related to the progress and outcomes of the KF-NAP and KF-PAT implementation at the research team, who summarized and shared the information periodically to lead therapists and their supervisors of all campuses
**Engaging** (Attracting and involving appropriate individuals in the implementation and use of the intervention through a combined strategy of social marketing, education, role modeling, training, and other similar activities). No barrier was identified in two sub-constructs including **formally appointed internal implementation leaders** (individuals from within the organization who have been formally appointed with responsibility for implementing an intervention as coordinator, project manager, team leader, or other similar roles) and **champions** (individuals who dedicate themselves to supporting, marketing, and driving through an implementation, overcoming indifference or resistance that the intervention may provoke in an organization).		
**Opinion leaders** (Individuals in an organization who have formal or informal influence on the attitudes and beliefs of their colleagues with respect to implementing the intervention)	• KF-NAP assessment: No barrier identified • KF-PAT treatment: ° Hesitance of some neuro-optometrists who were unfamiliar with the intervention.	• KF-NAP assessment: Not applicable • KF-PAT treatment: Invitations to neuro-optometrists for discussing the treatment mechanisms
**External change agents** (Individuals who are affiliated with an outside entity who formally influence or facilitate intervention decisions in a desirable direction)	• Length of stay may be shorter than anticipated	• Therapists were instructed to start the treatment very soon after admission, to be able to have as much treatment as possible provided

*This qualitative analysis was based on verbal reports and informal observations during (rather than after) the implementation process. Each identified barrier may represent one therapist's experience, a few participating site's situation, or a general observation of almost all participating sites. ADL, activities of daily living; IRF, inpatient rehabilitation facility; OT, occupational therapist*.

### Implementation Outcomes

We evaluated the implementation outcomes using quantitative and qualitative information that was collected during the 4-year period. The study also included collecting patient outcomes, which was reported separately (36, 37).

#### Numbers of Therapists Trained and Patients Receiving the Care

We used the number of OTs trained to indicate the success of knowledge dissemination and the number of patients receiving spatial neglect care through either the KF-NAP or KF-PAT to indicate the success of knowledge implementation. In April 2020, the research team asked the lead OTs to report the number of therapists they trained on both protocols, and what competency level they reached. In November 2020, we completed the collection of de-identified clinical records shared by participating sites. Note that patient outcomes (i.e., improvement in spatial neglect and rehabilitation outcomes) were reported in separate articles (36, 37).

Lead OTs were interviewed to provide context of the number of therapists trained and the number of patients receiving spatial neglect care. Responses to the interviews were summarized in writing with no audio records. Interview responses were reviewed by Authors PC and CGS.

#### User Feedback Survey

After the last monthly call (September 2020), a survey was sent to the lead OTs via an online platform, Survey Gizmo. The OTs were asked to report on the IRF that they represented, based on their own experience. See the survey in Appendix 1. The goal was to identify any sustaining barriers and additional facilitators to implementing either protocol now that the research team stepped back. Categorical responses were summarized and described in percentages. The qualitative answers were reviewed by Authors KH, PC, and CGS to enable the identification of emerged categories. We each separately read the data to determine reoccurring information, discussed and compared notes via conference calls and came to an agreement on how to code the data into similar groups or categories.

## Results

### Number of Therapists Trained

Table 3 reports that overall, 169 OTs at the participating sites were trained to some level of the KF-NAP, and among them, 81 (47.9%) reached the highest level (level 3) of competency. One hundred forty-one OTs were trained for the KF-PAT, and 110 (78.0%) reached the highest level of competency. Site 9 reported that they only implemented the KF-NAP at the beginning of the project and decided not to continue because an ongoing study was using the CBS following the original non-standardized questionnaire format (38). Nonetheless, Site 9 continued using the KF-PAT in their care.

**Table 3:** Participating rehabilitation hospitals and occupational therapists (OTs) trained.

**Site ID**	**Location (State)**	**Trained on site**	**Number of patients assessed using the KF-NAP^®^**	**Number of patients with spatial neglect**	**Number of patients treated with the KF-PAT^®^ for at least one session**	**Number of OTs trained (over the period from June 2017 to April 2020)**								**Lead OT change rate (number trained divided by number of therapists who left)**	**Monthly call attendance rate**
						**Competency level of the KF-NAP** ^**®**^				**Competency level of the KF-PAT** ^**®**^					
						Any level*	**1**	**2**	**3**	Any level*	**1**	**2**	**3**		
1	NJ	x	1,002	610	168	17	0	10	7	17	0	0	17	100%	73%
2	NJ	x	528	314	149	12	1	8	3	12	0	0	12	50%	73%
3	NJ	x	856	276	31	32	0	2	9	14	1	3	6	0%	73%
4	NJ	x	647	466	240	23	0	4	20	22	0	3	19	100%	86%
5	OH		82	79	30	6	0	0	6	4	0	0	4	100%	73%
6	PA		666	294	195	10	0	0	10	9	0	1	8	0%	86%
7	FL	x	239	77	64	6	0	2	1	3	0	2	1	0%	70%
8	MO		159	129	51	13	0	11	2	13	0	0	13	0%	81%
9	NY		17	16	14	4	0	0	4	4	0	0	4	100%	62%
10	MD	x	55	54	41	9	0	3	6	9	0	3	6	0%	91%
11	AZ		41	37	30	9	0	6	3	9	0	6	3	100%	95%
12	CA		13	11	6	2	0	0	2	2	0	0	2	67%	91%
13	GA	x	29	29	14	5	0	5	0	5	0	0	5	50%	78%
14	TX		49	29	9	6	1	4	1	3	2	0	1	0%	79%
15	OH		67	66	34	11	2	4	5	13	3	3	7	50%	47%
16	OH		4	4	2	4	2	0	2	2	0	0	2	0%	100%
Total		7	4,454	2,491	1,078	169	6	59	81	141	6	21	110	45% (average)	79% (average)

Sites that were not trained on site sent lead OTs to training sites. *Any level trained included therapists at level 1, 2, or 3 and therapists whose competency records were not available.

### Number of Patients Receiving Care

OTs assessed a total of 4,454 patients for spatial neglect using the KF-NAP, and 2,491 (56%) of them had the syndrome. 1,078 (43%) of the patients with spatial neglect were treated using the KF-PAT for at least one session.

To understand why more than half of the patients with spatial neglect did not receive PAT, we had discussions with the lead OTs during the conference calls and reviewed the OT's field notes in the de-identified clinical records in order to determine categories. Also, while the COVID pandemic led to a long pause in 2020 of no treatment using the KF-PAT Portable Kits across all participating sites, there were various factors that contributed to different implementation rates of either protocol in different sites, and therefore We classified the 15 participating sites (excluding Site 9) into three categories: early adopters, additional trained staff needed, and developing facilities.

Five sites (Sites 1, 2, 3, 4, and 6) were the first sites to be trained (aka early adopters). Therefore, many staff OTs, in addition to the implementation champions (lead OTs), were trained during the project. Although the rate of lead OT changes was 50% among these first trained sites, the other OTs were able to step up and take on the lead OT roles. These five sites collectively assessed 3,698 (83%) of all assessed patients and treated 783 (73%) of all treated patients across 16 sites. Based on the shared clinical records, the median CBS scores of PAT-untreated patients at these sites ranged from 2.5 to 3.75, which is a mild level of severity. In the de-identified clinical records and field notes, OTs reported that other deficits such as upper extremity impairment, rather than spatial neglect, was prioritized in OT sessions because the neglect was not severe.

Eight sites (Sites 5, 7, 8, 10, 12, 13, 15, and 16) were classified as “additional trained staff needed.” The median CBS scores of PAT-untreated patients at these sites ranged from 7.5 to 17.38, which is a wide range covering all levels of neglect severity. These sites relied on their lead OTs to implement both protocols while other OTs may or may not have integrated either protocol in their practice. Thus, only patients under lead OTs' care were assessed for spatial neglect, and other OTs who were not trained on the KF-NAP themselves, had to refer patients with neglect signs on their caseloads to the lead OTs for KF-NAP assessment. This indicated that patients whose neglect symptoms were less apparent may have not been identified and therefore not referred. Furthermore, not all patients who were confirmed with spatial neglect received PAT because the work load was too high for the lead OTs. Sites reported in field notes: “we do not have time to train other therapists in these protocols because of other responsibilities.” In addition, when a lead OT left, there was a lag in time during the transition and thus the average 45% change rate of implementation champions among these sites became a significant barrier. These eight sites planned to have additional staff trained early in Year 2020, which however was soon on pause due to the COVID pandemic. The pandemic caused particular challenges for Sites 15 and 16 who had joined the project a few months prior to the shutdown of research activities.

Lastly, four sites fell into the category of “developing facilities.” The management team was at the early stage of development in Sites 15 and 16 (also classified as “additional trained staff needed”) and Sites 11 and 14. These sites were having a hospital-wide staffing call, in order to recruit more therapist to work at their hospitals. Therefore, the combination of situations resulted in low numbers of identified individuals with neglect. That is, the infra-structure was not ready to fully support the implementation project.

### User Feedback Survey

**Figure 2:**
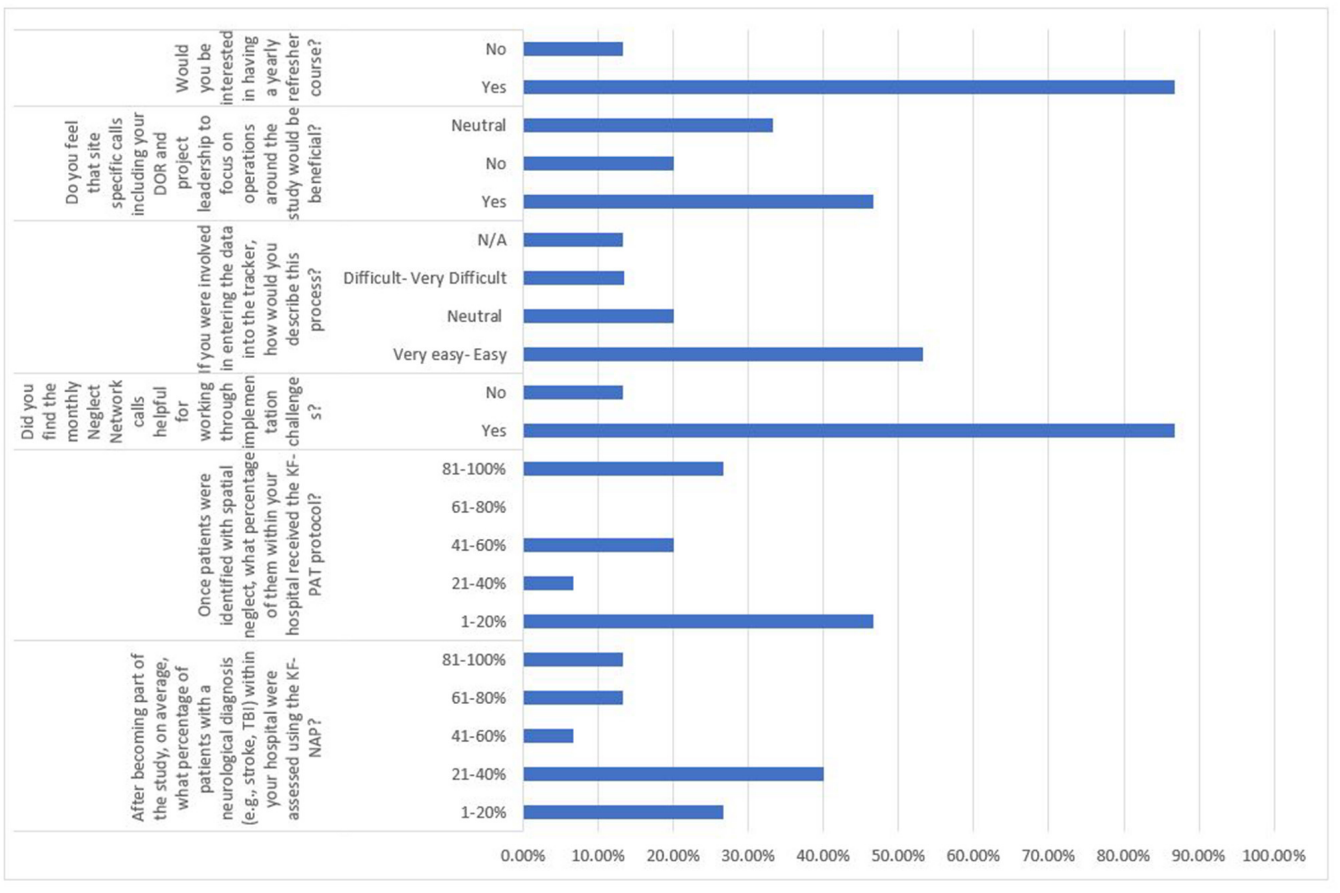
Summary of quantitative survey results in percentage.

Fifteen sites (93.75%) responded to the User Feedback survey. The quantitative aspect of the survey results was summarized in Figure 2. Multiple responses to open-ended questions were able to be categorized. First, the top three barriers related to the KF-NAP not being administered 100% of the time were: (1) lack of time to train the rest of staff on unit to perform the assessment, (2) patients being discharged earlier than expected, and (3) patients requiring other considerations at discharge such as extensive family training. It was also reported by multiple individuals that patients at multiple sites did not always receive all 10 KF-PAT sessions because of three reasons, categorized as: (1) short length of stay, (2) other clinical goals being prioritized, and (3) a lack of trained staff to assist with carrying out all 10 sessions. Besides the implementation of the two protocols, responses on the other aspects of the project included categories: (1) time consuming research documentation, (2) helpful monthly conference calls, and (3) supportive leadership.

## Discussion

There is still work to be done related to translating research into practice and decreasing the research-to-practice gap in the rehabilitation settings despite many efforts being made by many teams (26, 28). The shorter time it takes for the latest evidence to be applied to clinical practice, the greater chance for patients to receive better care (39). This manuscript highlights an implementation project that used the KTA cycle as the process model to assist the transfer of scientific knowledge into clinical practice. Specifically, the KTA cycle helped to guide the project development, report the results and provide specific information for future reproducibility. The CFIR was another implementation tool that was used in this project to assist with the categorization of barriers identified as well as provided a way to organize strategies that were trialed. Similar to others conducting rehabilitation implementation research (26), we found the use of the frameworks to be a strength of the study because the researcher-clinician team had a “road map” to guide implementation of the protocols as well as evaluate outcomes of the implementation.

The close collaboration between researchers and clinicians was key to achieve knowledge translation. The research team and the OTs had frequent communications through the de-identified clinical records and the monthly conference calls. This suggests that rather than a one-way, top-down instruction provision from the research team to the OTs, participants worked together and modified certain aspects on how to administer the KF-NAP and KF-PAT at specific sites. This is one example of how we used the KTA cycle and made an adaptation to fit the local context. However, it was important to the researchers that the core elements of both protocols remained unchanged, in other words, fidelity was maintained. For instance, regarding the KF-NAP protocol, as long as the therapist assessed tasks that relate to skin care or hair care (including facial) then they could be creative to what they ask the patient to complete (e.g., applying makeup instead of washing their face, which is the task suggested in the manual).

Most barriers identified during the implementation process were aligned with previous studies that offered reasons for the difficulties in knowledge translation and evidence-based practice (EBP) implementation in stroke care and rehabilitation (16, 40). One barrier, however, was unexpected. This was when consulting neuro-optometrists questioned whether administering PAT was within the scope of OT practice. The strategy addressing this barrier was to be collaborative, transparent, and be open to inter-professional learning. More specifically, the research team-initiated discussions with the neuro-optometrists about the mechanisms of prism adaptation and offered treatment demonstrations to share the procedures of the KF-PAT. Both KF-NAP and KF-PAT protocols were in use as part of clinical practice by the end of the implementation period. This suggests the tools were accepted by the OTs and adopted into the standard of care (41).

Another interesting finding was that focusing on a single discipline has the potential to change the overall quality of care in the multidisciplinary inpatient rehabilitation system. Shown in a prior study, only 31% of spatial neglect cases were mentioned in care team meetings, which potentially impeded provision of comprehensive care to all patients with spatial neglect (42). In the present project, the change of OT practice via the implementation of both spatial neglect assessment and treatment protocols increased the awareness of spatial neglect care among other disciplines. The CBS scores via the KF-NAP (indicating severity of spatial neglect) and improvements observed after PAT were discussed during care team meetings attended by all disciplines that provided medical and therapy services. Other disciplines could observe the procedures of both protocols easily as OTs worked in the same space with them. Thus, a new vocabulary was created and understood by all the care team members. This became an inner-setting facilitator (22) that emerged during the implementation process, different from facilitators provided by the research team and hospital administrative leaderships. Thus, the researcher-clinician collaboration is critical to initiate knowledge translation, and clinician buy-in and subsequent spontaneous inter-disciplinary communications are essential to strengthen the translation.

The therapists' time to complete the assessment and treatment was a limitation and was the most reported barrier. For the KF-NAP, the difficulty was related to the second (i.e., “post-treatment”) assessment, which is essential in order to measure changes in spatial neglect severity from before to after treatment. The second assessment which was to occur before the patients' discharge was difficult to administer when scheduling conflicts occurred more often at discharge than at admission. Time limitation with the assessment also affected treatment delivery. As recommended by the KF-PAT protocol, patients should complete the full treatment course which includes 10 sessions. In the present project, OTs at several sites shared that they prioritized other therapy activities over PAT when knowing that there was insufficient time to provide the recommended 10 sessions of PAT. This is due to the fact that the length of stay is usually estimated and pre-determined by insurers. Extending approved length of stay in an inpatient rehab solely for the purpose of completing the KF-NAP protocol may be applicable for a small percentage of patients with private insurers. For patients covered by Medicare's prospective payment plan, the hospital may elect to extent a patient's length of stay if the interdisciplinary team feels the additional days to complete the protocol would outweigh benefits of the projected discharge date. Among 2019 Medicare beneficiaries, for example, the average length of stay is 17, 16, and 15 days for patients with stroke, traumatic brain injury, and non-traumatic brain injury, respectively. Even if patients are assessed using the KF-NAP within the first few days of admission, there may be <10 full treatment days for a therapist to provide the recommended 10 once-daily PAT sessions. This factor in addition to many other factors may contribute to a decision not to provide PAT to certain patients.

The limited number of trained OTs in either protocol was another major barrier. This seems inconsistent with the fact that about 170 therapists were trained to integrate the KF-NAP and about 140 therapists were trained to use the KF-PAT in their practice across 16 IRFs. However, 10 participating sites primarily relied on lead OTs in administering the protocols, Who were unable to train other OTs due to time constraints, other clinical duties, and factors related to readiness of certain hospitals. The overall 45% change rate of lead OTs created lags of researcher-clinician communication and further slowed down the implementation progress. Regarding user feedback, 46.7% of survey respondents reported that it would be beneficial to have meetings or calls with their individual leadership, so they could share any continued barriers and determine strategies together. Almost 90% of the survey respondents mentioned that they would be interested in a yearly refresher course, on both protocols. This may help sustain implementation and also ensure fidelity (43). Thus, after collectively providing spatial neglect care to more than 4,500 patients in the context of this implementation project, OTs saw the need of continuing implementing KF-NAP and KF-PAT in their practice.

### Study Limitations

The project was initially driven by researchers and fueled by a collaborative effort shared by researchers and clinicians (including hospital administrative leaders). This is a strength but also the limitation of the project such that the outcomes may not be generalizable to facilities that have little access to researchers, especially researchers knowledgeable about implementation science. Another limitation was the inability to evaluate which strategies that were used toward eliminating a given barrier during the implementation process, had the best success rates vs. other strategies. It was not our priority to determine the best strategy but to offer solutions at the time when a barrier was present. Therefore, we cannot comment on the recommended strategies or which strategies should be trialed first. Further investigations formally testing outcomes such as feasibility, adoption and acceptability of delivering the protocols are needed to identify all the challenges to maintaining the implementation, and to determine how to overcome those challenges (43).

## Conclusion

The project demonstrated a researcher-clinician partnership in not only knowledge generation but also knowledge translation (e.g., dissemination and implementation of knowledge to be applied clinically). Evidence-based protocols can be implemented through multiple, tireless iterations of barrier reduction and problem solving with active participation of practitioners and practical support from leaders. There were no unintended consequences of the implementation efforts. Frequent communications and exchanging information with stakeholders at different levels, may be determinant to the success of each implementation phase. The results of the present project appeared promising in EBP implementation for spatial neglect care. However, further efforts are needed to promote the persistent inclusion of EBP for spatial neglect as the standard of care in inpatient rehabilitation. We also suggest the following future implementation efforts: (1) a pre-trial consultation with organizational leadership could ensure that sufficient clinician time can be blocked out, and (2) enabling all staff to receive training and deliver the intervention with higher levels of fidelity.

## Data Availability Statement

The data is not available to be shared.

## Ethics Statement

The studies involving human participants were reviewed and approved by Kessler Foundation. Written informed consent for participation was not required for this study in accordance with the national legislation and the institutional requirements.

## Author Contributions

KH and PC designed the study, analyzed and interpreted the data, and then wrote the manuscript. PC, KH, and CG-S interpreted the qualitative component of the study. JM contributed to editing the manuscript and was essential in data collection efforts. CG-S was essential to study member recruitment and edits to the entire manuscript. AB was key to study design and edited the entire manuscript. RG edited the manuscript. All authors read and approved the final manuscript.

## Funding

This work was supported by the Wallerstein Foundation for Geriatric Improvement and the Charles and Ann Serraino Foundation. Contents in this paper do not represent the policy of the funding agencies.

## Conflict of Interest

The KF-NAP and KF-PAT are registered trademarks of Kessler Foundation in the United States. JM and PC are employees of Kessler Foundation. The remaining authors declare that the research was conducted in the absence of any commercial or financial relationships that could be construed as a potential conflict of interest.

## Publisher's Note

All claims expressed in this article are solely those of the authors and do not necessarily represent those of their affiliated organizations, or those of the publisher, the editors and the reviewers. Any product that may be evaluated in this article, or claim that may be made by its manufacturer, is not guaranteed or endorsed by the publisher.

## Footnotes

^1^KF-PAT Portable Kit, Stoelting, 620 Wheat Lane, Wood Dale, IL, USA 60191.

## References

[1] Heilman KM Valenstein E Watson RT. Neglect and related disorders. Semin Neurol. (2000) 20:463–70. 10.1055/s-2000-13179 11149702

[2] Esposito E Shekhtman G Chen P. Prevalence of spatial neglect post-stroke: a systematic review. Ann Phys Rehabil Med. (2021) 64:101459. 10.1016/j.rehab.2020.10.010 33246185

[3] Chen P Ward I Khan U Liu Y Hreha K. Spatial neglect hinders success of inpatient rehabilitation in individuals with traumatic brain injury: a retrospective study. Neurorehabil Neural Repair. (2016) 30:451–60. 10.1177/1545968315604397 26338431

[4] Buxbaum LJ Ferraro MK Veramonti T Farne A Whyte J Ladavas E. Hemispatial neglect subtypes, neuroanatomy, and disability. Neurology. (2004) 62:749–56. 10.1212/01.WNL.0000113730.73031.F4 15007125

[5] Chen P Fyffe DC Hreha K. Informal caregivers' burden and stress in caring for stroke survivors with spatial neglect: an exploratory mixed-method study. Top Stroke Rehabil. (2017) 24:24–33. 10.1080/10749357.2016.1186373 27216085

[6] Chen P Hreha K Kong Y Barrett AM. Impact of spatial neglect on stroke rehabilitation: evidence from the setting of an inpatient rehabilitation facility. Arch Phys Med Rehabil. (2015) 96:1458–66. 10.1016/j.apmr.2015.03.019 25862254 PMC4519421

[7] Nijboer TCW Kollen BJ Kwakkel G. The impact of recovery of visuo-spatial neglect on motor recovery of the upper paretic limb after stroke. PLoS ONE. (2014) 9:e100584. 10.1371/journal.pone.0100584 24950224 PMC4065089

[8] Oh-Park M Hung C Chen P Barrett AM. Severity of spatial neglect during acute inpatient rehabilitation predicts community mobility after stroke. PMR. (2014) 6:716–22. 10.1016/j.pmrj.2014.01.002 24412266 PMC4090300

[9] Wee JYM Hopman WM. Comparing consequences of right and left unilateral neglect in a stroke rehabilitation population. Am J Phys Med Rehabil. (2008) 87:910–20. 10.1097/PHM.0b013e31818a58bd 18936556

[10] Yoshida T Mizuno K Miyamoto A Kondo K Liu M. Influence of right versus left unilateral spatial neglect on the functional recovery after rehabilitation in sub-acute stroke patients. Neuropsychol Rehabil. (2020) 1–22. 10.1080/09602011.2020.1798255. [Epub ahead of print]. 32703088

[11] Navarro M-D Lloréns R Noé E Ferri J Alcañiz M. Validation of a low-cost virtual reality system for training street-crossing. A comparative study in healthy, neglected and non-neglected stroke individuals. Neuropsychol Rehabil. (2013) 23:597–618. 10.1080/09602011.2013.806269 23767963

[12] Winstein CJ Stein J Arena R Bates B Cherney LR Cramer SC. Guidelines for adult stroke rehabilitation and recovery: a guideline for healthcare professionals from the American heart association/American stroke association. Stroke. (2016) 47:e98–e169. 10.1161/STR.0000000000000098 27145936

[13] Hebert D Lindsay MP McIntyre A Kirton A Rumney PG Bagg S. Canadian stroke best practice recommendations: stroke rehabilitation practice guidelines, update 2015. Int J Stroke Off J Int Stroke Soc. (2016) 11:459–84. 10.1177/1747493016643553 27079654

[14] Australian Clinical Practice Guidelines-Stroke Rehabilitation,. Stroke Foundation. (2021). Available online at: https://files.magicapp.org/guideline/11009797-9966-4e79-9344-afdf363f3503/published_guideline_5020-6_2.pdf (accessed December 18, 2021).

[15] Chen P Pitteri M Gillen G Ayyala H. Ask the experts how to treat individuals with spatial neglect: a survey study. Disabil Rehabil. (2018) 40:2677–91. 10.1080/09638288.2017.1347720 28697652

[16] Petzold A Korner-Bitensky N Salbach NM Ahmed S Menon A Ogourtsova T. Determining the barriers and facilitators to adopting best practices in the management of poststroke unilateral spatial neglect: results of a qualitative study. Top Stroke Rehabil. (2014) 21:228–36. 10.1310/tsr2103-228 24985390

[17] Bayley MT Hurdowar A Richards CL Korner-Bitensky N Wood-Dauphinee S Eng JJ. Barriers to implementation of stroke rehabilitation evidence: findings from a multi-site pilot project. Disabil Rehabil. (2012) 34:1633–8. 10.3109/09638288.2012.656790 22631218

[18] Fillion B Rochette A Girard A. Challenges of being a scholarly clinician as perceived by stroke rehabilitation professionals. Disabil Rehabil. (2014) 36:521–8. 10.3109/09638288.2013.797516 23721495

[19] Powell BJ Waltz TJ Chinman MJ Damschroder LJ Smith JL Matthieu MM. A refined compilation of implementation strategies: results from the expert recommendations for implementing change (ERIC) project. Implement Sci IS. (2015) 10:21. 10.1186/s13012-015-0209-1 25889199 PMC4328074

[20] Graham ID Logan J Harrison MB Straus SE Tetroe J Caswell W. Lost in knowledge translation: time for a map? J Contin Educ Health Prof. (2006) 26:13–24. 10.1002/chp.47 16557505

[21] Janzen S McIntyre A Richardson M Britt E Teasell R. Building a knowledge to action program in stroke rehabilitation. Can J Neurol Sci J Can Sci Neurol. (2016) 43:619–25. 10.1017/cjn.2016.258 27456566

[22] Damschroder LJ Aron DC Keith RE Kirsh SR Alexander JA Lowery JC. Fostering implementation of health services research findings into practice: a consolidated framework for advancing implementation science. Implement Sci. (2009) 4:50. 10.1186/1748-5908-4-50 19664226 PMC2736161

[23] Grol R Grimshaw J. From best evidence to best practice: effective implementation of change in patients' care. Lancet Lond Engl. (2003) 362:1225–30. 10.1016/S0140-6736(03)14546-1 14568747

[24] Powell BJ Fernandez ME Williams NJ Aarons GA Beidas RS Lewis CC. Enhancing the impact of implementation strategies in healthcare: a research agenda. Front Public Health. (2019) 7:1–9. 10.3389/fpubh.2019.00003 30723713 PMC6350272

[25] Powell BJ Proctor EK Glass JE. A systematic review of strategies for implementing empirically supported mental health interventions. Res Soc Work Pract. (2014) 24:192–212. 10.1177/1049731513505778 24791131 PMC4002057

[26] Leavy B Joseph C Kwak L Franzén E. Implementation of highly challenging balance training for Parkinson's disease in clinical practice: a process evaluation. BMC Geriatr. (2021) 21:96. 10.1186/s12877-021-02031-1 33526031 PMC7852138

[27] Pastva AM Coyle PC Coleman SW Radman MD Taylor KM Jones SB. Movement matters, and so does context: lessons learned from multisite implementation of the movement matters activity program for stroke in the comprehensive postacute stroke services study. Arch Phys Med Rehabil. (2021) 102:532–42. 10.1016/j.apmr.2020.09.386 33263286

[28] Bowden MG Monsch ED Middleton A Daughtry C Powell T Kraft SV. Lessons learned: the difficulties of incorporating intensity principles into inpatient stroke rehabilitation. Arch Rehabil Res Clin Transl. (2020) 2:100052. 10.1016/j.arrct.2020.100052 33543079 PMC7853341

[29] Chen P Hreha K. KF-NAP 2015 Manual. Kessler Foundation Learning Center. (2015). Available online at: https://www.kflearn.org/courses/kf-nap-2015-manuals

[30] Chen P Hreha K. KF-PAT 2020 Manual. Stoeling. Available online at: https://www.kflearn.org/courses/kf-pat-2018-manual (accessed April 21, 2020).

[31] Chen P Chen CC Hreha K Goedert KM Barrett AM. Kessler foundation neglect assessment process uniquely measures spatial neglect during activities of daily living. Arch Phys Med Rehabil. (2015) 96:869–76.e1. 10.1016/j.apmr.2014.10.023 25461827 PMC4410062

[32] Chen P Hreha K Fortis P Goedert KM Barrett AM. Functional assessment of spatial neglect: a review of the catherine bergego scale and an introduction of the kessler foundation neglect assessment process. Top Stroke Rehabil. (2012) 19:423–35. 10.1310/tsr1905-423 22982830 PMC3445290

[33] Fortis P Chen P Goedert KM Barrett AM. Effects of prism adaptation on motor-intentional spatial bias in neglect. Neuroreport. (2011) 22:700–5. 10.1097/WNR.0b013e32834a3e20 21817924 PMC3165096

[34] Chen P Goedert KM Shah P Foundas AL Barrett AM. Integrity of medial temporal structures may predict better improvement of spatial neglect with prism adaptation treatment. Brain Imag Behav. (2014) 8:346–58. 10.1007/s11682-012-9200-5 22941243 PMC3683116

[35] Goedert KM Chen P Foundas AL Barrett AM. Frontal lesions predict response to prism adaptation treatment in spatial neglect: a randomised controlled study. Neuropsychol Rehabil. (2018) 30:1–22. 10.1080/09602011.2018.1448287 29558241 PMC6148387

[36] Chen P Hreha K Gonzales-Snyder C Rich T Gillen R Barrett AM. Effects of prism adaptation treatment on spatial neglect and rehabilitation outcome: dosage matters. 10.1177/15459683221107891 35673990

[37] Chen P Diaz-Segarra N Hreha K Kaplan E Barrett AM. Prism adaptation treatment improves inpatient rehabilitation outcome in individuals with spatial neglect: a retrospective matched control study. Arch Rehabil Res Clin Transl. (2021) 3:100130. 10.1016/j.arrct.2021.100130 34589681 PMC8463461

[38] Azouvi P. Functional consequences and awareness of unilateral neglect: study of an evaluation scale. Neuropsychol Rehabil. (1996) 6:133–50. 10.1080/713755501

[39] Juckett LA Robinson ML Wengerd LR. Narrowing the gap: an implementation science research agenda for the occupational therapy profession. Am J Occup Ther. (2019) 73:7305347010p1–6. 10.5014/ajot.2019.033902 31484036

[40] Purvis T Moss K Denisenko S Bladin C Cadilhac DA. Implementation of evidence-based stroke care: enablers, barriers, and the role of facilitators. J Multidiscip Healthc. (2014) 7:389–400. 10.2147/JMDH.S67348 25246799 PMC4168868

[41] Proctor EK. Leverage points for the implementation of evidence-based practice. Brief Treat Crisis Interv. (2004) 4:227–42. 10.1093/brief-treatment/mhh02017636378

[42] Chen P McKenna C Kutlik AM Frisina PG. Interdisciplinary communication in inpatient rehabilitation facility: evidence of under-documentation of spatial neglect after stroke. Disabil Rehabil. (2013) 35:1033–8. 10.3109/09638288.2012.717585 23072734 PMC3660412

[43] Proctor E Silmere H Raghavan R Hovmand P Aarons G Bunger A. Outcomes for implementation research: conceptual distinctions, measurement challenges, and research agenda. Adm Policy Ment Health. (2011) 38:65–76. 10.1007/s10488-010-0319-7 20957426 PMC3068522

