# Supplementary material for: The Implementation Process of Two Evidence-Based Protocols: A Spatial Neglect Network Initiative
Source: Front Health Serv. 2022 Jun 23;2:839517. doi: 10.3389/frhs.2022.839517 (PMC10012810; doi:10.3389/frhs.2022.839517)
Supplement: Supplementary file 1 [file Data_Sheet_1.DOCX]

Appendix 1. Survey

|  | Question | Available answers |
| --- | --- | --- |
| 1 | What is the name of your hospital? | Open ended response |
| 2 | After becoming part of the study, on average, what percentage of patients with neurological diagnosis (e.g., stroke, traumatic brain injury) within your hospital were assessed using the KF-NAP | 1-20% |
|  |  | 21-40% |
|  |  | 41-60% |
|  |  | 61-80% |
|  |  | 81-100% |
| 3 | Once patients were identified with spatial neglect, what percentage of them within your hospital received the KF-PAT protocol? | 1-20% |
|  |  | 21-40% |
|  |  | 41-60% |
|  |  | 61-80% |
|  |  | 81-100% |
| 4 | Including yourself, if applicable, how many therapists from your hospital were initially trained to participate in this study? | 1 |
|  |  | 2 |
|  |  | >3 |
| 5 | For the initial training, did the therapist(s) travel to become trained or did someone train staff at your hospital? | Traveled for training, with other sites |
|  |  | Traveled to the trainer’s site for individual training |
|  |  | Staff were trained at my hospital by the trainer |
| 6 | How many additional therapists at your site were trained to conduct the KF-NAP after the initial training? | Open ended response |
| 7 | How many additional therapists at your site were trained to conduct the KF-PAT after the initial training? | Open ended response |
| 8 | In addition to staff support, what were the barriers to administering the KF-NAP to all neuro patients at admission and again at discharge? If applicable, please share 1-3 barriers. | Open ended response |
| 9 | Was the number of therapists trained to administer the KF-NAP or provide KF-PAT a barrier in implementing the tools more widely within your hospital? | Significant barrier |
|  |  | Barrier |
|  |  | Somewhat of a barrier |
|  |  | Not a barrier |
| 10 | If the number of therapists trained was a barrier, please describe what prohibited your site from training additional therapists. | Open ended response |
| 11 | Did the time involved to set up the equipment prohibit you from providing the KF-PAT? | Significant barrier |
|  |  | Barrier |
|  |  | Somewhat of a barrier |
|  |  | Not a barrier |
| 12 | Did the time involved for cleaning the equipment prohibit you from providing the KF-PAT? | Yes |
|  |  | No |
| 13 | Before the pandemic, do you believe other clinical needs of patients were prioritized over and prohibited the use of the KF-PAT? | Strongly agree |
|  |  | Agree |
|  |  | Disagree |
|  |  | Strongly disagree |
| 14 | On average, if the KF-PAT was provided, did patients receive all 10 treatments? | Yes |
|  |  | No |
| 15 | Please describe the primary reasons why patients with spatial neglect did not receive any or all 10 treatments. | Open ended response |
| 16 | If you were involved in entering the data into the tracker, how would you describe this process? | Very easy |
|  |  | Easy |
|  |  | Neutral |
|  |  | Difficult |
|  |  | Very Difficult |
|  |  | N/A |
| 17 | Did you find the monthly Neglect Network calls helpful for working through implementation challenges? | Occasionally helpful |
|  |  | Neutral |
|  |  | Somewhat helpful |
|  |  | Very helpful |
| 18 | Do you feel that it would be beneficial to have site specific calls which include your Director of Rehab and project leadership, to focus on operations around the study? | Strongly disagree |
|  |  | Disagree |
|  |  | Neutral |
|  |  | Agree |
|  |  | Strongly agree |
| 19 | Do you think the KF-NAP video-based tutorial is helpful when training new therapists? | Very helpful |
|  |  | Helpful |
|  |  | Somewhat helpful |
| 20 | Would you be interested in having a yearly refresher course? | Yes |
|  |  | No |
| 21 | Please provide any feedback (positive and/or constructive), challenges or concerns you have regarding the Spatial Neglect study | Open ended response |

Notes: KF-NAP = Kessler Foundation Neglect Assessment Process; KF-PAT = Kessler Foundation Prism Adaptation Treatment
